# A selenium species in cerebrospinal fluid predicts conversion to Alzheimer’s dementia in persons with mild cognitive impairment

**DOI:** 10.1186/s13195-017-0323-1

**Published:** 2017-12-19

**Authors:** Marco Vinceti, Annalisa Chiari, Marcel Eichmüller, Kenneth J. Rothman, Tommaso Filippini, Carlotta Malagoli, Jennifer Weuve, Manuela Tondelli, Giovanna Zamboni, Paolo F. Nichelli, Bernhard Michalke

**Affiliations:** 1 CREAGEN—Environmental, Genetic, and Nutritional Epidemiology Research Center, Department of Biomedical, Metabolic, and Neural Sciences, University of Modena and Reggio Emilia, 287 Via Campi, Modena, 41125 Italy; 2 Center for Neurosciences and Neurotechnology, Department of Biomedical, Metabolic, and Neural Sciences, University of Modena and Reggio Emilia, 287 Via Campi, Modena, 41125 Italy; 3 Department of Epidemiology, Boston University School of Public Health, 715 Albany Street, Boston, MA 02118 USA; 4 Department of Neurosciences, Azienda Ospedaliero-Universitaria di Modena, via del Pozzo 71, Modena, Italy; 5 Helmholtz Zentrum München GmbH—German Research Center for Environmental Health GmbH, Research Unit Analytical BioGeoChemistry, 1 Ingolstaedter Landstrasse, Neuherberg, 85764 Germany; 6 Research Triangle Institute, Research Triangle Park, 3040 E Cornwallis Road, Durham, NC 27709 USA

**Keywords:** Mild cognitive impairment, Alzheimer’s disease, Dementia, Selenium, Selenium species, Cerebrospinal fluid

## Abstract

**Background:**

Little is known about factors influencing progression from mild cognitive impairment to Alzheimer’s dementia. A potential role of environmental chemicals and specifically of selenium, a trace element of nutritional and toxicological relevance, has been suggested. Epidemiologic studies of selenium are lacking, however, with the exception of a recent randomized trial based on an organic selenium form.

**Methods:**

We determined concentrations of selenium species in cerebrospinal fluid sampled at diagnosis in 56 participants with mild cognitive impairment of nonvascular origin. We then investigated the relation of these concentrations to subsequent conversion from mild cognitive impairment to Alzheimer’s dementia.

**Results:**

Twenty-one out of the 56 subjects developed Alzheimer’s dementia during a median follow-up of 42 months; four subjects developed frontotemporal dementia and two patients Lewy body dementia. In a Cox proportional hazards model adjusting for age, sex, duration of sample storage, and education, an inorganic selenium form, selenate, showed a strong association with Alzheimer’s dementia risk, with an adjusted hazard ratio of 3.1 (95% confidence interval 1.0–9.5) in subjects having a cerebrospinal fluid content above the median level, compared with those with lower concentration. The hazard ratio of Alzheimer’s dementia showed little departure from unity for all other inorganic and organic selenium species. These associations were similar in analyses that measured exposure on a continuous scale, and also after excluding individuals who converted to Alzheimer’s dementia at the beginning of the follow-up.

**Conclusions:**

These results indicate that higher amounts of a potentially toxic inorganic selenium form in cerebrospinal fluid may predict conversion from mild cognitive impairment to Alzheimer’s dementia.

**Electronic supplementary material:**

The online version of this article (doi:10.1186/s13195-017-0323-1) contains supplementary material, which is available to authorized users.

## Background

Neurodegenerative dementias are well-recognized, severe medical conditions that are prevalent worldwide and expected to rise in western countries in the coming years [1, 2]. Effective therapies are lacking, as is adequate knowledge of their risk factors. In addition to genetic susceptibility, there is increasing evidence that environmental determinants, including environmental pollutants [3, 4], are important in dementia etiology. Among the large number of chemical factors that have been implicated in the etiology of dementia, particularly its most common form, Alzheimer’s dementia (AD), recent concern has focused on both increased and decreased exposure to the metalloid selenium (Se), an element of strong nutritional and toxicological interest [5–8]. Se exists in several chemical species with markedly different and even opposite biological properties [9–11]. In its selenocysteine-bound organic form, Se is an indispensable component in selenoprotein biosynthesis [7, 12], while other organic species such as selenomethionine-bound Se [13, 14] and the inorganic forms such as selenate or selenite [11, 15–17] are also well recognized as powerful toxicants. Se exposure in the human mainly occurs through diet and in its organic forms, its major sources being meat and fish, cereals, eggs, and dairy products [18, 19]. Se has been a topic of interest in recent decades mainly with reference to its possible role in cancer prevention and therapy [20–22]. More recently, its involvement in human brain pathology, and particularly with the risk of amyotrophic lateral sclerosis and AD, has become a focus [17, 23–29]. Such a relation, however, may exist only for some Se species, and particularly for the inorganic ones [30, 31].

However, while the results of a randomized trial assessing the effect of selenomethionine supplementation in dementia prevention have been published recently [32], there are no observational cohort studies specifically taking into account the speciation of the different chemical forms of Se in relation to the risk of AD.

We collected cerebrospinal fluid (CSF) samples in a cohort of Italian participants diagnosed with mild cognitive impairment (MCI) [33]. These participants were then followed for occurrence of dementia, enabling us to assess the relation between concentrations of various Se species in CSF and the risk of conversion to AD or other dementia.

## Methods

### Study cohort

**Fig. 1:**
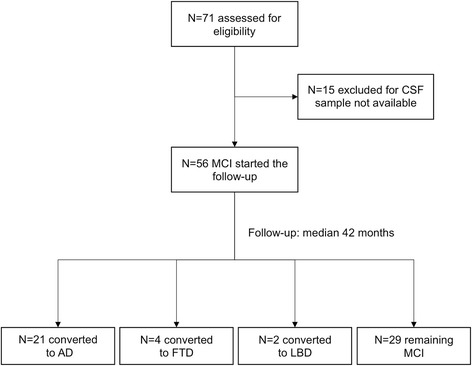
Flowchart for design of the cohort study. AD Alzheimer’s dementia, CSF cerebrospinal fluid, FTD frontotemporal dementia, LBD Lewy body dementia, MCI mild cognitive impairment

As shown in the flowchart (Fig. 1), following approval by the Modena Ethics Committee, we considered as eligible for our cohort study all participants who received a clinical diagnosis of MCI (amnestic MCI, single domain or multiple domain, or nonamnestic MCI [34, 35]) and who were admitted from 2008 to 2014 to the Neurology Memory Clinic of Sant’Agostino-Estense Hospital of Modena, Italy. Participants were then further selected if, following informed consent, they underwent a lumbar puncture (LP) for diagnostic purposes and had no brain imaging abnormalities or medical history suggestive of a vascular origin of their cognitive impairment [33, 36, 37]. Out of 71 potentially eligible participants, 56 had 1 mL of CSF or more available, and these constituted the cohort for the present study.

At baseline, all participants underwent routine blood tests, neurological and neuropsychological examination, and brain MRI. They also had a LP (within 1 month of clinical and neuropsychological examination) to measure CSF levels of Aβ_1–42_ (β-amyloid), total tau (t-tau), and phosphorylated tau (p-tau) proteins. APOE ε4 allele status was determined for 39 participants. All participants were subsequently followed up every 6 months through December 2016. At each visit they were classified according to whether their condition was stable or had converted to any clinical type of dementia, including AD [38], Lewy body dementia (LBD) [39], and frontotemporal dementia (FTD) [40, 41].

### Analytical determinations

Lumbar punctures were performed using a standard procedure to minimize the risk of biological and chemical contamination [17]. We collected CSF in sterile polypropylene tubes, which were transported to the adjacent laboratory within 30 min of collection. We centrifuged CSF for 15 min at 2700 × *g* at controlled room temperature and aliquoted into polypropylene storage tubes. CSF β-amyloid, t-tau, and p-tau 181 were measured as described previously [33]. The remaining, anonymized aliquots were immediately stored at – 80 °C and later transported deep frozen in dry ice by air courier to the element speciation laboratory at the Helmholtz Zentrum München, and kept continuously frozen until use.

We determined total Se by inductively coupled plasma dynamic reaction cell mass spectrometry (ICP-DRC-MS) and the Se species—selenite (Se(IV)), selenate (Se(VI)), selenomethionine-bound Se (Se-Met), selenocysteine-bound Se (Se-Cys), thioredoxin reductase-bound Se (Se-TXNRD), glutathione-peroxidase-bound Se (Se-GPX), selenoprotein P-bound Se (Se-SelenoP), and human serum albumin-bound Se (Se-HSA)—in CSF samples using ion exchange chromatography (IEC) coupled with ICP-DRC-MS, using methodologies we developed specifically for CSF [42, 43]. For total Se determination, CSF samples were diluted 1/10 with Milli-Q water + Rh as internal standard (1 μg/L final Rh concentration). For Se speciation, a Knauer 1100 Smartline inert Series gradient HPLC system was connected to an ion exchange column AS-11 (250 mm × 4 mm ID) from Thermo Fischer Scientific Inc. (Sunnyvale, CA, USA) for species separation. The sample volume (undiluted CSF) was 20 μl. Samples were determined in duplicate. The mobile phase consisted of eluent A (3.33 mM Tris–HAc buffer, 5% methanol, pH 8.0) and eluent B (10 mM Tris–HAc buffer, 500 mM ammonium acetate, 5% methanol, pH 8.0). Gradient elution was as follows: 0–3 min 100% eluent A (0% eluent B); 3–10 min 100–60% eluent A; 10–23 min 60–45% eluent A; 23–26 min 45–43% eluent A; 26–28 min 43–0% eluent A; 28–52 min 0% eluent A; 52–60 min 100% eluent A. The flow rate was constant at 0.8 mL/min. The column effluent was directed to ICP-DRC-MS. The experimental settings for ICP-DRC-MS (NexIon 300 D; Perkin Elmer) were: radiofrequency power 1250 W; plasma gas flow 15 L Ar/min; auxiliary gas flow 1.05 L Ar/min; nebulizer gas flow 0.92 L Ar/min; daily optimized dwell time 300 ms; ions monitored ^77^Se, ^78^Se, ^80^Se, and ^103^Rh (^103^Rh was used as internal standard for total Se determination); DRC reaction gas CH_4_ reaction at 0.58 mL/min; and DRC rejection parameter *q* 0.6. Five-point calibration curves with calibration points at 100, 500, 1000, 2000, and 5000 ng Se/L were linear with *r* ^2^ for the three Se isotopes being better than 0.999881. Data files from Se chromatograms were processed with Peakfit™ software for peak area integration.

Concerning the analytical figures of merit and analytical quality control, the limit of detection (LOD) was 19.5 ng Se/L for Se species. Only values above the LOD are reported throughout the manuscript. Accuracy of Se determination and Se species quantification was checked by analyzing control materials and a certified reference material: quality control for total Se determination was performed by analyzing control materials ‘human serum’ and ‘urine’ from Recipe (Munich, Germany). Control materials were reconstituted as indicated on the flask labels. The resulting solutions were diluted 1/50 (serum, measurement concentration 1.25 μg/L) or 1/10 (urine, measurement concentration 2.35 μg/L) with Milli-Q water before measurements for adjusting the measurement concentration to the expected concentration range of CSF (no CSF reference material for Se was available). Accuracy values were 98.4 ± 3.8% (serum) and 102.1 ± 5.4% (urine).

The certified reference material NIST 1950 (National Institute of Standards and Technology, Gaithersburgh, MD, USA) was used for quality control regarding total Se, Se-SelenoP, Se-GPX, and Se-HSA. Accuracy values were 103 ± 5.1% (Se-SelenoP, target value = 100%: 50.2 ± 4.3 μg/kg), 93 ± 3.1% (Se-GPX, target value = 100%: 23.6 ± 1.3 μg/kg), and 97 ± 1.7% (Se-HSA, target value = 100%: 28.2 ± 2.6 μg/kg).

### Data analysis

For analytical values below the LOD, we input half of the threshold [44, 45]. Most participants had Se species well above the LOD (from 89 to 100% depending on the single Se form), with lower values only for the three organic Se species Se-Cys, Se-GPX, and Se-TXNRD, which had values above the LOD only in 21%, 43%, and 0% of the participants, respectively. We assessed the association between Se species through Spearman correlation. To evaluate the possible influence of Se species on β-amyloid and p-tau, we also fitted a linear regression model of log-transformed CSF concentrations of β-amyloid and, separately, p-tau at baseline. In both the Spearman correlation analysis and the linear regression analysis, values of Se species below the LOD were excluded. After defining the person-time of follow-up as the time of MCI diagnosis/CSF sampling until the last follow-up visit, December 2016, or the date of AD/dementia diagnosis, whichever occurred first, we estimated the hazard ratio (HR) of progressing to AD (as well as to any dementia subtype, i.e., AD + FTD + LBD) in a Cox proportional hazards model. After assessing all variables for the proportional hazard assumption, we fitted a multivariable Cox model stratified by sex, and adjusted for age (years), education (years), and duration of sample storage (years).

## Results

Table 1 reports the main demographic and clinical characteristics of MCI cohort members at baseline according to dementia diagnosis during the follow-up, and Table 2 reports their CSF concentrations of Se species, β-amyloid, t-tau, and p-tau. Of the original 56 participants, 21 converted to AD, four to FTD, and two to LBD, and 29 did not convert at the end of the follow-up. Follow-up lasted on average 43.3 months, with a median of 42 months and an interquartile range of 30.4–51.2, with a total number of person-months of follow-up equal to 2423.5.

**Table 1:** Baseline characteristics of study population according to diagnosis at the end of follow-up

	MCI		AD		FTD		LBD	
	*N*	(%)	*N*	(%)	*N*	(%)	*N*	(%)
All participants	29	(100)	21	(100)	4	(100)	2	(100)
Sex								
Males	17	(58.6)	10	(47.6)	2	(50.0)	1	(50.0)
Females	12	(41.4)	11	(52.4)	2	(50.0)	1	(50.0)
Age at entry								
< 65 years	14	(48.3)	6	(28.6)	4	(100)	–	
≥ 65 years	15	(51.7)	15	(71.4)	–		2	(100)
Education								
< 8 years	11	(37.9)	5	(28.8)	–		2	(100)
8–12 years	8	(27.6)	8	(38.1)	4	(100)	–	
≥ 13 years	10	(34.5)	8	(38.1)	–		–	
APOE ɛ4 carriership								
Noncarriers	14	(48.3)	4	(19.0)	2	(50.0)	1	(50.0)
Carriers	8	(27.6)	9	(42.9)	–		1	(50.0)
Missing	7	(24.1)	8	(38.1)	2	(50.0)	–	
Months of follow-up^a^	44.4	(31.1–55.3)	35.5	(29.6–47.4)	43.1	–	38.5	–

*AD* Alzheimer’s dementia, *APOE* apolipoprotein E, *FTD* frontotemporal dementia, *LBD* Lewy body dementia, *MCI* mild cognitive impairment

^a^Median (interquartile range)

**Table 2:** Distribution of levels of selenium species and β-amyloid, t-tau, and p-tau at baseline in cerebrospinal fluid of the study population according to diagnosis at the end of follow-up

	MCI (*N* = 29)		AD (*N* = 21)		FTD (*N* = 4)	LBD (*N* = 2)
	50th percentile	IQR	50th percentile	IQR	50th percentile	50th percentile
Total Se (nmol/L)	51.67	(47.11–57.75)	55.72	(45.97–64.46)	47.87	59.27
Inorganic Se (nmol/L)	7.98	(5.83–9.50)	8.49	(5.45–10.13)	7.47	9.50
Se(IV)	5.19	(4.31–7.22)	5.07	(3.80–7.85)	6.46	8.49
Se(VI)	1.52	(1.14–3.93)	2.91	(1.65–4.31)	1.01	1.01
Organic Se (nmol/L)	23.81	(16.21–28.75)	20.26	(13.04–27.61)	18.24	29.89
Se-SelenoP	20.64	(15.20–25.84)	18.36	(11.90–23.18)	16.34	26.72
Se-Met	1.65	(1.01–2.79)	1.90	(0.89–2.91)	1.39	3.17
Se-Cys	0.13	(0.13–0.13)	0.13	(0.13–0.13)	0.13	0.13
Se-GPX	0.13	(0.13–1.14)	0.13	(0.13–0.76)	0.51	0.13
Se-HSA (nmol/L)	17.73	(14.69–22.67)	20.90	(14.69–23.30)	17.22	16.08
Unknown (nmol/L)	3.17	(1.77–4.56)	3.55	(2.03–5.95)	1.90	4.18
β-amyloid (pg/mL)	699	(521–963)	506	(417–519)	761	611
t-tau (pg/mL)	256	(198–404)	625	(404–743)	222	355
p-tau (pg/mL)	60	(46–85)	86	(73–128)	47	67

*AD* Alzheimer’s dementia, β*-amyloid* Aβ_1–42_, *FTD* frontotemporal dementia, *IQR* interquartile range, *LBD* Lewy body dementia, *MCI* mild cognitive impairment, *p-tau* phosphorylated tau protein, *Se* selenium, *Se(IV)* selenite, *Se(VI)* selenate, *Se-SelenoP* selenoprotein P-bound Se, *Se-Met* selenomethionine-bound Se, *Se-Cys* selenocysteine-bound Se, *Se-GPX* glutathione-peroxidase-bound Se, *Se-HSA* human serum albumin selenium-bound Se, *t-tau* total tau protein

When we assessed the correlation between Se species, the only forms not associated with total Se were Se(VI), Se-GPX, and Se-Cys, the latter form being also unrelated to total organic Se. Inorganic Se (particularly Se(VI)) was inversely correlated with organic Se and particularly Se-SelenoP and Se-Met. Se-HSA was directly correlated with all Se species and categories except for Se-Met, Se-Cys, and Se-GPX. However, all correlations involving Se-Cys and especially Se-GPX were based on a small number of individuals, because a large proportion of the sample concentrations fell below the LOD.

Inorganic Se and Se(VI) concentrations in baseline CSF samples were inversely associated with CSF β-amyloid concentration (Table 3). By contrast, higher organic Se concentration and particularly Se-Met in CSF were associated with higher CSF β-amyloid. There was little evidence of an association of any Se species in CSF with CSF concentration of p-tau; Se(VI) showed some evidence of a direct relation with this protein, although this was statistically unstable as shown by the wide confidence interval of the regression coefficient. Further adjustment for APOE ɛ4 allele carriership did not substantially change the results (Additional file 1: Table S1).

**Table 3:** Linear regression analysis of CSF selenium species levels versus log-transformed values of biomarkers of Alzheimer’s disease pathology (β-amyloid and p-tau as dependent variables) in the 56 MCI study participants at baseline

Se species	(*N*)	Crude		Adjusted	
		β	95% CI	β	95% CI
β*-amyloid*					
Total Se	(56)	0.03	(–0.09 to 0.15)	0.02	(–0.11 to 0.15)
Inorganic Se	(56)	–0.27	(–0.66 to 0.11)	–0.26	(–0.66 to 0.15)
Se(IV)	(53)	–0.12	(–0.75 to 0.51)	–0.09	(–0.78 to 0.60)
Se(VI)	(50)	–0.74	(–1.63 to 0.16)	–0.81	(–1.74 to 0.13)
Organic Se	(56)	0.16	(0.02 to 0.30)	0.18	(0.01 to 0.34)
Se-SelenoP	(56)	0.15	(–0.00 to 0.31)	0.17	(–0.02 to 0.36)
Se-Met	(54)	1.87	(0.52 to 3.23)	2.30	(0.81 to 3.79)
Se-Cys	(12)	1.01	(–3.17 to 5.18)	2.93	(–1.33 to 7.18)
Se-GPX	(24)	0.41	(–1.50 to 2.33)	0.52	(–1.60 to 2.64)
Se-HSA	(25)	–0.00	(–0.22 to 0.21)	0.02	(–0.22 to 0.25)
Unknown	(56)	–0.08	(–0.57 to 0.41)	–0.14	(–0.72 to 0.43)
*p-tau*					
Total Se	(56)	0.06	(–0.08 to 0.19)	0.09	(–0.05 to 0.23)
Inorganic Se	(56)	–0.02	(–0.45 to 0.41)	–0.09	(–0.53 to 0.35)
Se(IV)	(53)	–0.31	(–0.98 to 0.37)	–0.58	(–1.29 to 0.14)
Se(VI)	(50)	0.33	(–0.62 to 1.28)	0.23	(–0.74 to 1.20)
Organic Se	(56)	0.02	(–0.14 to 0.18)	0.10	(–0.08 to 0.28)
Se-SelenoP	(56)	0.03	(–0.15 to 0.20)	0.13	(–0.08 to 0.33)
Se-Met	(54)	–0.26	(–1.83 to 1.31)	0.10	(–1.63 to 1.82)
Se-Cys	(12)	–0.69	(–5.45 to 4.60)	–1.59	(–8.23 to 5.05)
Se-GPX	(24)	–0.09	(–2.52 to 2.34)	–0.45	(–3.11 to 2.21)
Se-HSA	(25)	0.05	(–0.20 to 0.30)	0.04	(–0.22 to 0.31)
Unknown	(56)	0.61	(0.10 to 1.12)	0.75	(0.17 to 1.33)

Adjusted estimates are from a multivariable model including sex, age, education, and duration of sample storage as potential confounders. Values below the limit of detection were excluded from the analysis

β*-amyloid* Aβ_1–42_, *CI* confidence interval, *CSF* cerebrospinal fluid, *MCI* mild cognitive impairment, *p-tau* phosphorylated tau protein, *Se* selenium, *Se(IV)* selenite, *Se(VI)* selenate, *Se-SelenoP* selenoprotein P-bound Se, *Se-Met* selenomethionine-bound Se, *Se-Cys* selenocysteine-bound Se, *Se-GPX* glutathione-peroxidase-bound Se, *Se-HSA* human serum albumin selenium-bound Se

Results of the proportional hazards regression analysis are reported in Table 4. We observed an excess, albeit statistically unstable, AD risk associated with higher total Se, with exposure classified into a dichotomy, above or below the median both in the crude analysis and taking into account age and sex, time elapsed since year of first storage, and education. When looking at the single Se species, we found a strongly increased AD risk associated with Se(VI) exposure and to a lesser extent with Se-Met, and more weakly with Se-HSA. Results were roughly comparable in the analysis based on continuous values of Se exposure (both one-unit and one-standard-deviation increase; data not shown). When we adjusted for β-amyloid and p-tau, in addition to age and sex, we obtained HRs comparable with those obtained in the less adjusted analysis, although in this adjusted model the excess risk associated with overall Se, organic Se, Se-SelenoP, and particularly Se-Met levels was enhanced, and that associated with Se(VI) was reduced. Finally, adding APOE ε4 status to the most adjusted multivariable model shown in Table 4 had little effect on the estimates (Additional file 1: Table S2), with the exception of the HR associated with selenate which became 7.6 (95% CI 1.2–49.5). However, results of the latter analysis were statistically less stable due to fewer participants (for a few cohort members this genetic datum was not available) and more variables in the model.

**Table 4:** Crude and adjusted HR of developing Alzheimer’s dementia in a Cox proportional hazards model, comparing participants with baseline selenium CSF concentrations above versus below (reference) the median value

Se species	Crude		Adjusted^a^		Adjusted^b^	
	HR	95% CI	HR	95% CI	HR	95% CI
Total Se	1.6	(0.7–3.9)	1.5	(0.6–3.8)	2.0	(0.7–5.9)
Inorganic Se	0.8	(0.3–1.9)	1.2	(0.5–3.0)	1.0	(0.4–2.9)
Se(IV)	0.6	(0.2–1.4)	0.7	(0.3–1.7)	0.8	(0.3–2.2)
Se(VI)	2.6	(1.0–6.7)	3.1	(1.0–9.5)	2.4	(0.7–7.8)
Organic Se	1.4	(0.6–3.3)	0.9	(0.3–2.4)	1.0	(0.3–2.8)
Se-SelenoP	1.3	(0.6–3.2)	0.9	(0.3–2.3)	1.0	(0.3–2.8)
Se-Met	1.3	(0.5–3.0)	1.2	(0.5–3.0)	2.3	(0.9–5.9)
Se-Cys	0.6	(0.2–1.9)	0.6	(0.2–1.9)	0.6	(0.2–2.2)
Se-GPX	1.1	(0.5–2.6)	1.0	(0.4–2.5)	0.8	(0.3–2.2)
Se-HSA	1.2	(0.5–2.9)	1.3	(0.5–3.5)	1.7	(0.5–5.3)
Unknown	1.0	(0.4–2.4)	2.7	(0.8–9.3)	4.1	(0.9–18.8)

*CI* confidence interval, *CSF* cerebrospinal fluid, *HR* hazard ratio, *MCI* mild cognitive impairment, *Se* selenium, *Se(IV)* selenite, *Se(VI)* selenate, *Se-SelenoP* selenoprotein P-bound Se, *Se-Met* selenomethionine-bound Se, *Se-Cys* selenocysteine-bound Se, *Se-GPX* glutathione-peroxidase-bound Se, *Se-HSA* human serum albumin selenium-bound Se

^a^Adjusted for sex, age at entry, years of storage, and years of education

^b^Adjusted for sex, age at entry, years of storage, years of education, and β-amyloid and phosphorylated tau protein level

When we replaced AD with any dementia occurrence in the aforementioned analyses, effect estimates were substantially unchanged (data not shown). We also repeated the aforementioned Cox analysis by omitting the AD cases detected after the first 2 years of follow-up (two participants): results were substantially unchanged, with the HR associated with selenate levels above the median being 3.5 (95% CI 1.0–11.9).

When we stratified the analysis according to the APOE ε4 status, a direct association of Se(VI) with AD risk emerged in both APOE ε4 categories (Additional file 1: Table S3), either in crude analyses or after adjusting for potential confounders. Considering the most adjusted model, in the 21 APOE ε4 noncarriers HRs were also increased for total Se, Se-GPX, and Se-HSA, while in the 18 APOE ε4 carriers an increased AD risk was apparent for total Se, Se-Met, and Se-Cys. Because of the small numbers within strata, however, these effect estimates were imprecise, as indicated by their wide confidence intervals.

## Discussion

We investigated whether the risk of conversion to AD in patients with MCI is influenced by exposure to Se. We found that one out of several Se species in CSF was positively associated with subsequent AD, and results were similar when we included in the outcome the few additional incident cases of neurodegenerative dementia; that is, the four cases of FTD and the two cases of LBD. The Se species associated with AD was the inorganic hexavalent one, selenate (Se(VI)), a species that lacks a direct physiological role by itself as it is not incorporated into selenoproteins. In addition, we found some associations between the organic form Se-Met and AD risk, but these were not confirmed in analyses based on the exclusion of cases diagnosed early during the follow-up.

Se(VI) is characterized by a peculiar metabolic pattern and toxicity [8, 11, 14, 24, 46–50]. Our findings, which were confirmed in analyses that take into account potential confounders such as education [51] and duration of sample storage [10], indicate that higher amounts of this Se species may predict and possibly cause AD. This association remained, but was weaker, after including in the multivariable model two biomarkers of Alzheimer’s disease pathology, β-amyloid and p-tau levels. This finding indicates that these two CSF proteomic indicators may be mediators of Se(VI) toxicity, in which case they should be omitted from the regression model. Some association remained when these factors were included in the model, which could indicate that these two factors may not mediate the entire possible neurodegenerative effect of Se(VI), or result from discrepancy in the CSF measures as indicators of neuropathology in the parenchyma. We also noted in the linear regression analysis an inverse association between baseline CSF Se(VI) (and more generally inorganic Se) and β-amyloid. This observation strengthens our findings from the Cox regression model, since low CSF β-amyloid levels are a marker of AD conversion risk in individuals with MCI [33]. The reason for such an inverse relation specifically restricted to inorganic Se and particularly to Se(VI) is difficult to surmise. It may be linked to some specific toxic properties such as pro-oxidant activity and promotion of protein misfolding by this Se species, as suggested or documented in laboratory and animal studies [16, 52–54] and suspected to occur in humans [8, 24]. It also may relate to specific genetic features in Se(VI) metabolism characterizing some individuals [49].

A deleterious effect of Se(VI) on the central nervous system (CNS), and more generally inorganic Se, is biologically plausible, since these Se compounds have been long known to be very toxic [24, 55]. Such an effect would contrast with suggestions of potential beneficial effects of Se and selenoproteins in AD progression from laboratory studies [56–58], although this hypothesis was recently contradicted by results from the PREADVISE study (Prevention of Alzheimer’s Disease by Vitamin E and Selenium Trial) [32]. In that trial, 7540 asymptomatic older adults in North America were randomized to either placebo or 200 μg/day Se as l-selenomethionine or both l-selenomethionine and vitamin E for an average of 5.4 years, but there was no effect of any Se supplementation on dementia or AD incidence during the active supplementation period, or within a subset of the study cohort up to 6 additional years [32]. In the laboratory and veterinary medicine studies, inorganic and some organic Se species were shown to disrupt physiological pathways related to the etiology of neurological disorders or induce frank neurotoxicity [24]. This is particularly true for inorganic Se [59–61] including Se(VI) [62, 63], which may induce oxidative stress [62, 64, 65] and cause genotoxicity and apoptosis [53, 66–69], particularly in neural cells [60]. This Se species may also be incorporated into protein as a replacement for sulfur, with consequent misfolding and functional impairment [65, 70] and endoplasmic reticulum stress [54, 71], all mechanisms potentially involved in AD etiology [72, 73]. In humans, specific neurotoxicity data are available for Se(VI) only for acute high-dose intoxication, which includes confusion, memory loss, anxiety, depression, irritability, insomnia, and dizziness [74, 75]. Exposure to inorganic Se species and Se(VI) in the human is limited, since Se(VI) levels in food are low compared with organic Se forms, and Se in drinking water (generally containing Se as Se(VI)) contributes little to total Se intake [76]. However, there are scarce data on Se speciation in food, and some sources such as seafood or Se-enriched vegetables may contain higher levels of the inorganic Se(VI) species [18, 77]. Dietary supplements may also represent a source of Se(VI), although they contain a mixture of Se species, in most cases represented mainly by its organic forms, especially Se-Met [78, 79].

The two key features of our study are the longitudinal design and the speciation approach. The cohort design allowed us to avoid reverse causality, the major potential limitation of certain case–control studies and all cross-sectional studies. In fact, a progressive deterioration of nutritional status may characterize progression to AD, in parallel with the worsening of cognitive impairment [29, 80], and selectively involve at an early stage of the disease some dietary factors including Se [81]. In addition, an effect of disease itself on Se tissue distribution and metabolism might exist. This effect is also suggested by the higher levels in post-mortem AD brains of the antioxidant SelenoP, which has been interpreted as a compensatory response to the oxidative stress characterizing disease progression [82]. Despite the strength of the longitudinal design of our study, we also took into account the possibility that some clinically undetected incipient disease already characterized our participants later converting to AD, and therefore that some possibility of reverse causation still existed. We addressed this point by removing participants developing AD in the first period of follow-up, and our associations did not change or were even strengthened.

Focusing on Se speciation is something that has not been done before in similar research. Earlier studies assessed only overall Se levels or, very rarely, selenoprotein activity [82, 83]. Since the chemical form of Se plays a major role in driving both its toxicological and nutritional effects, any exposure assessment based on overall Se content may be misleading [8, 9, 11, 15]. Also neurotoxic properties of various Se species may differ considerably, independently of the overall Se exposure [24, 84]. These considerations accentuate the potential for bias due to exposure misclassification based on overall Se determination in epidemiologic studies [10, 21, 22], and highlight the relevance of speciation analysis in neurodegeneration research [30].

Another important feature of this study is the investigation of Se status in a CNS compartment. In fact, peripheral biomarkers of exposure, either based on overall Se or on single Se species, may not adequately predict CNS levels, especially in view of the known peculiarities of metabolism and retention of this element in the brain [85, 86] and the lack of correlation between some circulating Se species, especially its inorganic forms, with CSF levels [15, 43, 87]. Most case–control studies of AD have focused on peripheral indicators of exposure, such as blood, urine, hair, and nail samples, finding conflicting results ranging from adverse to protective [27, 28], while little association was noted with Se CSF levels [88–90].

A recent study based on 286 autopsied samples found Se brain content to be positively associated with brain neuropathology [91]. Se content was directly and positively correlated with neurofibrillary tangle severity, and in the highest exposure category a higher but statistically unstable risk of global Alzheimer’s disease pathology and of Lewy bodies also emerged [91]. However, the cross-sectional study design and lack of speciation analyses made it impossible to assess whether the higher Se levels preceded brain neuropathology or were due to compensatory selenoprotein synthesis [7, 8, 92].

We also found an excess but statistically imprecise AD risk associated with Se-HSA. However, the interpretation of this finding is challenging because of uncertainties about the exact nature of this chemical species, which might include both organic and inorganic Se forms [10, 15]. Finally, we found some evidence of an excess AD risk associated with ‘unknown’ Se species, but the very wide confidence interval of the effect estimate and the uncertainties of the nature of this Se compound(s) hamper a reliable assessment of this finding.

We found some evidence of effect modification by the APOE ε4 status on AD risk, indicating that carriers of the APOE ε4 allele may undergo an excess disease risk for higher levels of Se-Met and Se-SelenoP. Interestingly, an indication that APOE ε4 status and Se metabolism may interact has been provided in an older Chinese population [93]. Se-Met is a Se form which has cytotoxic and pro-oxidant activities [69, 94], and this species has been recently observed to induce cognitive impairment in an animal model [95].

As already indicated, our study was small, with insufficient data to assess the associations within subgroups according to sex, age, or other factors. Similarly, we lacked data to assess the role of the three Se species (Se-Cys, Se-GPX, and Se-TXNRD) for which most samples (all for Se-TXNRD) fell below the LOD. Therefore, the involvement of these species in AD etiology could not be adequately or even partially assessed in the present study. Another limitation is the prospect of unmeasured confounding [96], which appears to be of particular relevance in epidemiologic studies dealing with Se [97]. Finally, the association between selenium as Se(VI) and AD risk found in our study may apply only to a population having the Se exposure typical of residents in the study area, already shown in previous studies to be comparable with the Italian national average [98–100], while such association may not necessarily exist in other populations characterized by considerably lower intake of this element [90, 101].

## Conclusions

We found in persons with mild cognitive impairment of nonvascular origin that a higher cerebrospinal fluid content of an inorganic Se species, selenate, predicted progression toward AD. No other Se form was related to either increased or increased AD risk. Since results were strengthened when participants who were diagnosed early during the follow-up were excluded from the analysis, thus limiting any effect of reverse causality, our results indicate that selenate levels in the central nervous system compartment may predict and possibly influence AD risk. However, the possibility of unmeasured confounding and the statistical imprecision of our results emphasize the need to replicate these findings in other studies.

## Additional file

Additional file 1: Table S1. presenting APOE ɛ4-adjusted linear regression analysis estimates of CSF Se species versus log-transformed values of biomarkers of Alzheimer’s disease pathology (β-amyloid and p-tau as dependent variables) in the MCI study participants at baseline (values below the limit of detection for each specific species excluded from analysis). Most-adjusted estimates are from a multivariable model including sex, age, education, and duration of sample storage as potential confounders, in addition to APOE ɛ4 allele carriership (presence/absence). **Table S2.** presenting crude and APOE ɛ4-adjusted HRs of developing AD in a Cox proportional hazards model according to baseline CSF Se species in the 39 MCI subjects at baseline having information about their APOE ɛ4 status. Se exposure status defined as 0 (below or equal) and 1 (above) with reference to the median value. **Table S3.** presenting crude and adjusted HRs of developing AD in a Cox proportional hazards model, related to baseline CSF Se species in MCI subjects at baseline, according to APOE ɛ4 carriership status (carriers, *N* = 18; noncarriers, *N* = 21). Se exposure status defined as 0 (below or equal) and 1 (above) with reference to the median value. (DOCX 29 kb)

## Abbreviations

AD: Alzheimer’s dementia
CI: Confidence interval
CSF: Cerebrospinal fluid
FTD: Frontotemporal dementia
HR: Hazard ratio
IQR: Interquartile range
LBD: Lewy body dementia
LOD: Limit of detection
MCI: Mild cognitive impairment
Se(IV): Selenite
Se(VI): Selenate
Se-Cys: Selenocysteine-bound Se
Se-GPX: Glutathione-peroxidase-bound Se
Se-HSA: Human serum albumin selenium-bound Se
Se-Met: Selenomethionine-bound Se
Se-SelenoP: Selenoprotein P-bound Se
Se-TXNRD: Thioredoxin reductase-bound Se
