# Supplementary material for: A selenium species in cerebrospinal fluid predicts conversion to Alzheimer’s dementia in persons with mild cognitive impairment
Source: Alzheimers Res Ther. 2017 Dec 19;9:100. doi: 10.1186/s13195-017-0323-1 (PMC5735937; doi:10.1186/s13195-017-0323-1)
Supplement: Additional file 1: Table S1. — presenting APOE ɛ4-adjusted linear regression analysis estimates of CSF Se species versus log-transformed values of biomarkers of Alzheimer’s disease pathology (β-amyloid and p-tau as dependent variables) in the MCI study participants at baseline (values below the limit of detection for each specific species excluded from analysis). Most-adjusted estimates are from a multivariable model including sex, age, education, and duration of sample storage as potential confounders, in addition to APOE ɛ4 allele carriership (presence/absence). Table S2. presenting crude and APOE ɛ4-adjusted HRs of developing AD in a Cox proportional hazards model according to baseline CSF Se species in the 39 MCI subjects at baseline having information about their APOE ɛ4 status. Se exposure status defined as 0 (below or equal) and 1 (above) with reference to the median value. Table S3. presenting crude and adjusted HRs of developing AD in a Cox proportional hazards model, related to baseline CSF Se species in MCI subjects at baseline, according to APOE ɛ4 carriership status (carriers, N = 18; noncarriers, N = 21). Se exposure status defined as 0 (below or equal) and 1 (above) with reference to the median value. (DOCX 29 kb) [file 13195_2017_323_MOESM1_ESM.docx]

**Table S1.** APOE ɛ4-adjusted linear regression analysis estimates of CSF Se species versus log-transformed values of biomarkers of Alzheimer’s disease pathology (β-amyloid and phosphorylated tau protein (p-tau) as dependent variables) in the MCI study participants at baseline (values below the limit of detection for each specific species excluded from analysis). Most-adjusted estimates are from a multivariable model including sex, age, education and duration of sample storage as potential confounders, in addition to APOE ɛ4 allele carriership (presence/absence).

|  |  | Only APOE ɛ4-adjusted | |  | Most-adjusted model (including APOE ɛ4) | |
| --- | --- | --- | --- | --- | --- | --- |
| Se species | (N) | β | 95% CI |  | β | 95% CI |
| *β-amyloid* |  |  |  |  |  |  |
| Total Se | (39) | 0.08 | (-0.06 to 0.23) |  | 0.07 | (-0.08 to 0.21) |
| Inorganic Se | (39) | -0.18 | (-0.63 to 0.28) |  | -0.22 | (-0.68 to 0.24) |
| Se(IV) | (37) | -0.18 | (-0.91 to 0.55) |  | -0.34 | (-1.13 to 0.45) |
| Se(VI) | (35) | -0.37 | (-1.74 to 0.39) |  | -0.81 | (-1.87 to 0.25) |
| Organic Se | (39) | 0.12 | (-0.03 to 0.27) |  | 0.15 | (-0.02 to 0.32) |
| Se-SelenoP | (39) | 0.10 | (-0.08 to 0.27) |  | 0.13 | (-0.07 to 0.33) |
| Se-Met | (39) | 1.95 | (0.55 to 3.34) |  | 2.61 | (1.16 to 4.06) |
| Se-Cys | (6) | 2.74 | (-6.44 to 11.92) |  | - | - |
| Se-GPX | (17) | 1.65 | (-0.68 to 3.99) |  | 2.37 | (-0.08 to 4.76) |
| Se-HSA | (38) | 0.13 | (-0.10 to 0.35) |  | 0.15 | (-0.08 to 0.38) |
| Unknown | (39) | 0.17 | (-0.37 to 0.72) |  | 0.01 | (-0.62 to 0.64) |
| *P-tau* |  |  |  |  |  |  |
| Total Se | (39) | 0.04 | (-0.12 to 0.21) |  | 0.08 | (-0.09 to 0.25) |
| Inorganic Se | (39) | -0.09 | (-0.61 to 0.44) |  | -0.11 | (-0.65 to 0.43) |
| Se(IV) | (37) | -0.27 | (-1.09 to 0.55) |  | -0.37 | (-1.26 to 0.52) |
| Se(VI) | (35) | 0.22 | (-0.98 to 1.41) |  | 0.11 | (-1.13 to 1.36) |
| Organic Se | (39) | 0.04 | (-0.14 to 0.22) |  | 0.13 | (-0.08 to 0.33) |
| Se-SelenoP | (39) | 0.06 | (-0.14 to 0.27) |  | 0.18 | (-0.05 to 0.41) |
| Se-Met | (39) | -0.54 | (-2.30 to 1.23) |  | -0.22 | (-2.22 to 1.78) |
| Se-Cys | (6) | -2.90 | (-8.81 to 3.02) |  | - | - |
| Se-GPX | (17) | -1.68 | (-5.00 to 1.63) |  | -1.56 | (-5.57 to 2.44) |
| Se-HSA | (38) | -0.01 | (-0.29 to 0.27) |  | -0.03 | (-0.33 to 0.27) |
| Unknown | (39) | 0.59 | (0.00 to 1.19) |  | 0.78 | (0.11 to 1.46) |

CI, confidence intervals; CSF, cerebrospinal fluid; MCI, mild cognitive impairment; Se(IV), selenite; Se(VI), selenate; Se-SelenoP, selenoprotein P-bound Se; Se-Met, selenomethionine-bound Se; Se-Cys, selenocysteine-bound Se; Se-GPX, glutathione-peroxidase-bound Se; Se-HSA, human serum albumin selenium-bound Se.

**Table S2.** Crude and APOE ɛ4-adjusted hazard ratio (HR) of developing AD in a Cox proportional hazards model according to baseline CSF Se species in the 39 MCI subjects at baseline having information about their APOE ɛ4 status. Selenium exposure status defined a 0 (below or equal) and 1 (above) with reference to the median value.

|  | Crude | |  | Adjusted^a^ | |  | Adjusted^b^ | |
| --- | --- | --- | --- | --- | --- | --- | --- | --- |
| Se species | HR | 95% CI |  | HR | 95% CI |  | HR | 95% CI |
| Total Se | 1.9 | (0.6 - 5.8) |  | 1.9 | (0.6 - 6.0) |  | 2.2 | (0.6 - 8.0) |
| Inorganic Se | 0.6 | (0.2 - 1.8) |  | 0.6 | (0.2 - 1.8) |  | 1.0 | (0.3 - 3.5) |
| Se(IV) | 0.5 | (0.2 - 1.6) |  | 0.6 | (0.2 - 1.9) |  | 0.7 | (0.2 - 2.6) |
| Se(VI) | 3.9 | (1.0 - 14.7) |  | 3.4 | (0.9 - 13.1) |  | 7.6 | (1.2 - 49.5) |
| Organic Se | 1.9 | (0.6 - 6.2) |  | 2.0 | (0.6 - 6.2) |  | 1.2 | (0.3 - 4.3) |
| Se-SelenoP | 2.0 | (0.6 - 6.4) |  | 2.1 | (0.7 - 6.5) |  | 1.3 | (0.3 - 4.7) |
| Se-Met | 0.9 | (0.3 – 2.6) |  | 1.2 | (0.4 – 3.9) |  | 1.0 | (0.3 - 3.6) |
| Se-Cys | 0.7 | (0.2 - 3.2) |  | 0.7 | (0.1 - 3.0) |  | 1.1 | (0.2 - 6.9) |
| Se-GPX | 1.3 | (0.4 - 3.9) |  | 1.1 | (0.4 - 3.3) |  | 1.2 | (0.4 - 3.8) |
| Se-HSA | 1.5 | (0.5 - 4.5) |  | 1.4 | (0.5 - 4.4) |  | 1.6 | (0.4 - 5.8) |
| Unknown | 1.5 | (0.4 - 5.8) |  | 1.6 | (0.4 - 5.9) |  | 7.3 | (1.3 - 41.1) |

^a^Adjusted for APOE status (ɛ4 allele carriership presence/absence).

^b^Adjusted for sex, age at entry, years of storage, years of education and APOE ɛ4 status.

AD, Alzheimer’s dementia; CI, confidence intervals; CSF, cerebrospinal fluid; HR, hazard ratio; MCI, mild cognitive impairment; Se(IV), selenite; Se(VI), selenate; Se-SelenoP, selenoprotein P-bound Se; Se-Met, selenomethionine-bound Se; Se-Cys, selenocysteine-bound Se; Se-GPX, glutathione-peroxidase-bound Se; Se-HSA, human serum albumin selenium-bound Se.

**Table S3**. Crude and adjusted Hazard Ratio (HR) of developing AD in a Cox proportional hazards model, related to baseline CSF Se species MCI subjects at baseline, according to APOE ɛ4 carriership (carriers, N=18; non-carriers, N=21) status. Selenium exposure status defined a 0 (below or equal) and 1 (above) with reference to the median value.

|  | Crude analysis | | | | |  | Adjusted analysis^a^ | | | | |  | Adjusted analysis^b^ | | | | |
| --- | --- | --- | --- | --- | --- | --- | --- | --- | --- | --- | --- | --- | --- | --- | --- | --- | --- |
|  | APOE ɛ4 non-carriers | |  | APOE ɛ4 carriers | |  | APOE ɛ4 non-carriers | |  | APOE ɛ4 carriers | |  | APOE ɛ4 non-carriers | |  | APOE ɛ4 carriers | |
| Se species | HR | 95% CI |  | HR | 95% CI |  | HR | 95% CI |  | HR | 95% CI |  | HR | 95% CI |  | HR | 95% CI |
| Total Se | 1.9 | (0.1 - 7.3) |  | 2.5 | (0.6 - 10.3) |  | 1.0 | (0.1 - 14.3) |  | 2.1 | (0.4 - 11.6) |  | 2.3 | (0.1 - 108.7) |  | 2.3 | (0.4 - 12.3) |
| Inorganic Se | 0.2 | (0.0 - 1.9) |  | 0.9 | (0.2 - 3.2) |  | 0.3 | (0.0 - 2.7) |  | 0.5 | (0.1 - 3.0) |  | 0.4 | (0.0 - 4.2) |  | 1.2 | (0.2 - 7.5) |
| Se(IV) | 0.0 | (0.0 - ---) |  | 1.4 | (0.4 - 5.2) |  | 0.0 | (0.0 - ---) |  | 1.0 | (0.2 - 4.6) |  | 0.0 | (0.0 - ---) |  | 1.6 | (0.2 - 9.7) |
| Se(VI) | 5.1 | (0.5 - 50.5) |  | 2.5 | (0.5 - 12.6) |  | 4.8 | (0.4 - 60.0) |  | 4.0 | (0.4 - 39.0) |  | 5.5 | (0.2 - 163.0) |  | 13.0 | (0.9 - 179.1) |
| Organic Se | 2.0 | (0.2 - 21.9) |  | 2.1 | (0.5 - 8.2) |  | 1.6 | (0.1 - 18.2) |  | 1.6 | (0.4 - 6.9) |  | 0.4 | (0.0 - 8.6) |  | 0.6 | (0.1 - 4.1) |
| Se-SelenoP | 2.0 | (0.2 - 21.9) |  | 2.3 | (0.6 - 9.0) |  | 1.6 | (0.1 - 18.2) |  | 1.8 | (0.4 - 7.4) |  | 0.4 | (0.0 - 8.6) |  | 0.9 | (0.1 - 6.3) |
| Se-Met | 0.6 | (0.1 - 4.2) |  | 1.7 | (0.5 - 6.5) |  | 0.3 | (0.0 - 3.9) |  | 5.3 | (0.7 - 38.5) |  | 0.3 | (0.0 - 5.4) |  | 2.3 | (0.3 - 16.8) |
| Se-Cys | 2.0 | (0.2 - 22.4) |  | 0.4 | (0.1 - 3.7) |  | 1.7 | (0.1 - 24.6) |  | 0.7 | (0.1 - 6.2) |  | 0.4 | (0.0 - 24.9) |  | 3.6 | (0.1 - 98.2) |
| Se-GPX | 5.2 | (0.4 - 68.3) |  | 0.8 | (0.2 - 3.0) |  | 31.5 | (0.1 - to high) |  | 1.0 | (0.3 - 4.2) |  | 29.8 | (0.0 - to high) |  | 1.2 | (0.2 - 5.5) |
| Se-HSA | 0.6 | (0.1 - 7.2) |  | 1.8 | (0.4 - 7.2) |  | 0.7 | (0.1 - 8.4) |  | 1.4 | (0.3 - 5.7) |  | 22.2 | (0.1 - to high) |  | 1.4 | (0.3 - 6.5) |
| Unknown | 1.0 | (0.1 - 12.9) |  | 1.8 | (0.4 - 8.6) |  | 1.2 | (0.1 - 22.4) |  | 1.3 | (0.2 - 7.7) |  | 30.3 | (0.1 - to high) |  | 19.3 | (1.2 - 378.3) |

^a^Adjusted for sex and age at entry.

^b^Adjusted for sex, age at entry, years of storage and years of education.

AD, Alzheimer’s dementia; CI, confidence intervals; CSF, cerebrospinal fluid; HR, hazard ratio; MCI, mild cognitive impairment; Se(IV), selenite; Se(VI), selenate; Se-SelenoP, selenoprotein P-bound Se; Se-Met, selenomethionine-bound Se; Se-Cys, selenocysteine-bound Se; Se-GPX, glutathione-peroxidase-bound Se; Se-HSA, human serum albumin selenium-bound Se.
